# The association between COVID-19 anxiety levels and tobacco use among patients within a smoking cessation polyclinic

**DOI:** 10.18332/tid/149180

**Published:** 2022-06-10

**Authors:** Sibel Baktır Altuntaş, Hilal Özkaya, Ahmet Beşel, Sümeyra Betül Namlı, Kübra Albayrak

**Affiliations:** 1 Department of Family Medicine, Başakşehir Çam and Sakura State Hospital, Istanbul, Turkey; 2 Department of Family Medicine, University of Health Sciences Gaziosmanpaşa Training and Research Hospital, Istanbul, Turkey

**Keywords:** smoking cessation, anxiety, COVID-19

## Abstract

**INTRODUCTION:**

The aim of this study was to investigate the impact of the COVID-19 pandemic on the anxiety level of smokers and the relationship between smoking behavior and COVID-19 anxiety level.

**METHODS:**

Our study was planned as a descriptive cross-sectional survey. A 32-question face-to-face questionnaire containing the Coronavirus Anxiety Scale (CAS) and the Fagerström test for nicotine dependence (FTND) was administered to 349 patients who had applied to the smoking cessation polyclinic between 15 May 2021 and 1 August 2021. SPSS 25.00 was used for statistical analysis.

**RESULTS:**

A total of 349 individuals participated in the study. The mean CAS and FTND total scores were 0.89±2.13 (range: 0–20) and 6.34±2.53 (range: 0–10), respectively. There was a statistically significant difference between CAS total score and gender (p=0.005), marital status (p=0.006), changes in the amount of smoking during the pandemic (p=0.011), and impact of the COVID-19 pandemic on smoking cessation (p<0.001). The impact of the COVID-19 pandemic on smoking cessation was statistically significantly different between knowing that the rate of intensive care unit (ICU) admissions due to COVID-19 infection is higher in smokers, smokers are heavily infected with COVID-19 and the mortality rate due to COVID-19 infection is higher in smokers (p<0.001).

**CONCLUSIONS:**

Anxiety due to the COVID-19 pandemic may cause a change in the amount of smoking and the decision to quit smoking. Healthcare professionals should transform COVID-19 anxiety into an opportunity to improve health and quit smoking, one of the healthy behaviors.

## INTRODUCTION

Smoking, which is designated as the most important preventable health problem by the World Health Organization (WHO), has been defined as a disorder in the ICD-11 table^1^. Smoking cessation polyclinics occupy a key position in the treatment of addiction to tobacco products, especially cigarettes, and in the provision of counselling services^2^. The importance of treating smoking addiction was reemphasized with the declaration of the COVID-19 epidemic as a pandemic on 11 March 2020^3^.

Additional risk factors in the course of COVID-19 infection, which can range from mild (fever, dry cough, myalgia) to severe (pneumonia) and life-threatening (respiratory failure, sepsis syndrome, multiple organ failure), are the forerunners of the severity of the disease^4^. Among the risk factors for COVID-19 associated pneumonia, the role of smoking is controversial. It has been previously suggested that high ACE-2 expression in smokers predisposes them to increased SARS-CoV-2 infection^5^. There are analyses confirming that the severity of COVID-19 disease is two times higher in smokers than never smokers^6^. Current evidence suggests that smoking is associated with increased disease severity and mortality in hospitalized COVID-19 patients^7^.

To reduce the risk of transmission in COVID-19 infections, which have a high mortality rate, many countries have adopted stringent measures such as physical distancing measures, restrictions on international travel, closure of schools, and postponement of meetings and demonstrations^8^. Despite the implementation of these non-pharmaceutical measures taken, rising infection and death rates and uncertainty about the future may induce high levels of emotional distress and avoidance behaviors in people who are very worried about contracting COVID-19^9^.

Somatic symptoms such as insomnia, loss of appetite, and dyspeptic symptoms often occur in conjunction with anxiety in response to extreme fear or worry. Anxiety due to COVID-19 is common, and the Coronavirus Anxiety Scale (CAS) was developed by Lee et al.^10^ to measure anxiety. The scale assesses symptoms of anxiety and dysfunctional thinking according to DSM-5 criteria^11^. The Turkish validity study of the questionnaire was conducted by Ladikli et al.^12^. It is a brief mental health screening to identify possible dysfunctional anxiety related to the COVID-19 crisis. Each item of the CAS measures the response to a different physiologically based fear or anxiety related to thoughts or information about coronavirus. The CAS examines five different somatically based fear and anxiety symptoms, including dizziness, tonic immobility, sleep disturbance, loss of appetite, nausea, or abdominal discomfort triggered by thoughts and information about the coronavirus^10^. While there are studies showing that fear and anxiety triggered by the pandemic increases adherence to protective measures (hygiene rules, mask, and social distance) to protect against infection, there are also studies showing that they contribute to the development of unhealthy behaviors such as increased alcohol consumption and decreased physical activity^13^. The impact of the pandemic on smoking behavior, which is one of the addictive substances, varies^14^.

This study aims to examine the impact of the COVID-19 pandemic on smokers’ anxiety levels and the relationship between smoking behavior and COVID-19 anxiety levels.

## METHODS

### Study population and sample

Our study was as a descriptive cross-sectional study and was conducted in the Smoking Cessation Polyclinic, between 31 May 2021 and 1 August 2021. Since the mean number of patients presenting to the smoking cessation polyclinic within one month between March and April was 160, the sample size was calculated to be 220 with a 95% confidence interval (n=220). Patients who applied to the outpatient clinic on the dates of the study were briefed about the study. Questionnaires were administered to those who met the inclusion criteria.

*Inclusion criteria*

The inclusion criteria were: patients presenting to the smoking cessation polyclinic for the first time; the date of first application of patients who had previously presented to the smoking cessation polyclinic should not be before May 31; no history of using psychiatric drugs (within the last 6 months); no psychiatric illness should have been diagnosed; no memory problems and no memory-related neurological disease (Alzheimer’s disease, vascular dementia, Lewy body dementia and frontotemporal dementia, mixed dementia); the ability to speak, understand and read Turkish; not to be pregnant; and be aged >18 years.

### Data collection tools

The questionnaire form used in the study was prepared by the researchers. A total of 22 questions were asked. Questions reported on the sociodemographic characteristics of the participants and the frequency of use of tobacco and tobacco products, their smoking behavior during the COVID-19 pandemic, the time to decide to quit smoking, the impact of COVID -19 pandemic on smoking cessation, the status of having a COVID-19 infection, the presence of individuals who had COVID-19 in their immediate surroundings, and information questions about the COVID-19 infection progress in smokers. Survey questions also included the questions of the Coronavirus Anxiety Scale (CAS) and the Fagerström test for nicotine dependence (FTND). The CAS is used to determine the frequency of anxiety symptoms experienced by individuals, consisting of 5 questions whose Turkish validity for measuring anxiety levels was performed^10,12^. Each item of the CAS was scored on a 5-point Linkert scale ranging from 0 (never) to 4 (almost every day) based on experience in the past two weeks. The minimum score for each question was 0, and the maximum score was 4. The total score ranged from 0–20, and higher scores meant that the coronavirus-related anxiety was greater.

The Fagerström test for nicotine dependence (FTND) is the most common test designed to assess tobacco dependence. The reliability and validity study of FTND was conducted in Turkey and found to be applicable in Smoking Cessation Clinics^15^. FTND has a graded scale (0–10) corresponding to the degree of nicotine dependence. Those who scored 0–2 points are considered to have a nicotine dependence that is ‘very mild’, 3–4 points ‘mild’, 5 points ‘moderate’, 6–7 points ‘severe’, and 8–10 points ‘extremely severe’.

### Ethical considerations

Approval was obtained from the Ethics Committee of Başakşehir Çam and Sakura City Hospital for this study and was conducted in accordance with the principles of the Declaration of Helsinki. Participation in the study was on a voluntary basis, and informed consent was obtained from each participant. Confidentiality and anonymity of the respondents were also ensured.

### Statistical analysis

All statistical analyses were performed using IBM SPSS version 25.0 (SPSS Inc., Chicago, Illinois USA). Continuous variables are presented as mean±SD, and some of the categorical variables are given as frequency (n) and percentage (%). In addition, Cronbach’s alpha internal consistency coefficients were calculated by employing the results of participants’ minimum, maximum, and mean scores on the scales. Parametric tests were used in the study because the number of participants exceeded 200^16^. Pearson correlation analysis was performed to determine the relationship between the total scores of the scales. Independent samples t-test and one-way ANOVA, which are parametric tests, were applied to determine if there was a significant difference between the independent variables and the total scores of the scales. In case of significant difference between the groups, the *post hoc* test was conducted to determine between which groups the significance was. The Sidak *post hoc* test was selected due to homogeneous variance distribution and unequal number of samples^17^. The chi-squared test was used to compare categorical variables. Multivariate linear regression analysis was performed to determine the variables predicting CAS total score. A p<0.05 was considered statistically significant.

## RESULTS

### Sociodemographic characteristics of the respondents

A total of 349 subjects participated in the study. The mean age of the participants was 36.8±10.7; 35.5% (n=124) were female. While the proportion of those whose tobacco use increased during the pandemic COVID-19 was 26.1% (n=91), 10% (n=35) decreased their tobacco use. The sociodemographic data are shown in Table 1.

**Table 1:** Sociodemographic characteristics of the participants (N=349)

*Characteristics*	*n or Mean (range)*	*% or Mean±SD*
**Gender**		
Female	124	35.5
Male	225	64.5
**Age** (years)	36.0 (18.0–78.0)	36.79±10.74
≤20	11	3.2
21–30	106	30.4
31–40	108	30.9
41–50	92	26.4
>50	32	9.2
**Education level**		
Primary school or literate	88	25.2
Middle/high school	166	47.6
Undergraduate degree	84	24.1
Postgraduate degree	11	3.2
**Profession**		
Officer	41	11.7
Employee	139	39.8
Self-employed	82	23.5
Retired	17	4.9
Housewife	61	17.5
Other	9	2.6
**Marital status**		
Married	240	68.8
Single	90	25.8
Widowed/divorced	19	5.4
**Chronic disease**		
Present	83	23.8
Absent	266	76.2
**Regular use of medication**		
Present	103	29.5
Absent	246	70.5
**Are there any COVID-19 positive people around you?**		
Yes	161	46.1
No	165	47.3
I’m not sure	23	6.6
**If there is a COVID-19 positive person around you, where?**		
At home in my family	78	48.4
At the workplace	42	26.1
Other	41	25.5
**Have you had a COVID-19 infection?**		
Yes	35	10.0
No	295	84.5
I’m not sure	19	5.4
**Were you hospitalized when you contracted the COVID-19 infection?**		
Yes	1	2.9
No, I was treated at home	34	97.1
Which tobacco and tobacco products do you use? * (n=370)		
Cigarette	347	93.8
Hookah	16	4.3
Electronic cigarette	7	1.9
**How long have you been smoking?** (years)	17.0 (1.0–65.0)	18.26±10.31
0–10	110	31.5
11–20	123	35.2
21–30	75	21.5
>30	41	11.7
**The amount of smoking during the pandemic**		
Increased	91	26.1
Decreased	35	10.0
Unchanged	223	63.9
**When did you decide to quit smoking?**		
Before the epidemic began	182	52.1
When the epidemic began	23	6.6
After the epidemic	144	41.3
**Do you think the COVID-19 pandemic has an impact on smoking cessation?**		
Yes	131	37.5
No	218	62.5
**Smokers are heavily infected with COVID-19**		
Wrong/no answer	142	40.7
Right	207	59.3
**The rate of intensive care unit (ICU) admissions due to COVID-19 infection is higher in smokers**		
Wrong/no answer	137	39.3
Right	212	60.7
**The mortality rate due to COVID-19 infection is higher among smokers**		
Wrong/no answer	135	38.7
Right	214	61.3
**Nicotine dependence according to FTND**		
Very mild	32	9.2
Mild	48	13.8
Moderate	41	11.7
Severe	99	28.4
Extremely severe	129	37.0

* The number n exceeds the sample size because there were multiple responses. FTND: Fagerström test for nicotine dependence.

### Comparison of the CAS and FTND for the different variables

The internal consistency coefficients of Cronbach’s alpha for the scales used in the questionnaire ranged from 0.66–0.80. Since these values were between or above the recommended limits of 0.50–0.70 in the literature, the internal consistency of the scales in this study was adequate^18-20^.

The mean CAS total score was 0.89±2.13 (range: 0–20) and FTND total score obtained in the questionnaire was 6.3±2.5 (range: 0–10), while no statistically significant correlation was found between the FTND total score and the CAS total score (r=0.046, p=0.389).

Table 2 presents the analysis of results regarding the comparison of CAS and FTND total scores with different variables. The CAS was higher in women than in men (1.4±2.8 vs 0.6±1.6, p=0.005). CAS total mean scores were higher in widowed/divorced (7.16±2.09) than in married (6.06±2.57) or single (6.94±2.40) (p=0.006). The Coronavirus Anxiety Scale total score and the change in the amount of smoking during the pandemic were compared. The mean score of CAS was higher in those who reported that the amount of smoking decreased (20.88±13.27) than in those who reported that it increased (13.00±10.68) or did not change (17.64±10.64) (p=0.011). Finally, the mean score of the CAS was higher among those who thought the pandemic had an impact (1.60±2.96) than among those who said it had no impact on their thinking about quitting smoking (0.46±1.27) (p<0.001). According to this analysis, a statistically significant difference was found between the FTND total score and age (p=0.037) and changes in the amount of smoking during the pandemic (p<0.001).

**Table 2:** Comparison of the CAS and FTND scores for the different variables (N=349)

*Variables*	*Categories*	*n*	*FTND total score*	*t/F*	*p*	*CAS total score*	*t/F*	*p*
			*Mean±SD*			*Mean±SD*		
**Gender**	Male	124	6.52±2.54	0.927	0.354	1.40±2.79	2.868	**0.005**
	Female	225	6.25±2.53			0.61±1.62		
**Age** (years)	≤20	11	7.18±2.40	2.586	**0.037**	1.64±2.91	1.498	0.202
	21–30	106	6.80±2.33			0.79±1.79		
	31–40	108	6.38±2.58			1.21±2.66		
	41–50	92	5.98±2.69			0.66±1.73		
	>50	32	5.50±2.34			0.53±1.93		
**Education level**	Primary school or literate	88	6.64±2.54	0.771	0.511	1.01±2.00	0.730	0.535
	Middle/high school	166	6.31±2.46			0.75±1.94		
	Undergraduate degree	84	6.20±2.62			1.10±2.67		
	Postgraduate degree	11	5.64±2.91			0.45±1.51		
**Marital status**	Married	240	0.88±2.17	0.040	0.961	6.06±2.57	5.155	**0.006**
	Single	90	0.94±2.07			6.94±2.40		
	Widowed/divorced	19	0.84±2.19			7.16±2.09		
**Chronic disease**	Present	83	1.00±2.24	0.531	0.596	6.20±2.69	-0.584	0.560
	Absent	266	0.86±2.11			6.39±2.49		
**Regular use of medication**	Present	103	0.87±1.95	-0.098	0.922	6.19±2.63	-0.727	0.467
	Absent	246	0.90±2.22			6.41±2.49		
**Are there any COVID-19 positive people around you?**	Yes	161	1.11±2.31	1.755	0.174	6.29±2.47	0.716	0.490
	No	165	0.67±1.73			6.32±2.54		
	I'm not sure	23	1.00±3.23			6.96±2.93		
**If there is a COVID-19 positive person around you, where? (n=161)**	At home in my family	78	0.90±2.13	1.884	0.155	5.88±2.52	2.098	0.126
	At the workplace Other	42 41	0.90±2.28			6.71±2.48		
	Other	41	1.71±2.61			6.63±2.29		
**Have you had a COVID-19 infection?**	Yes	35	5.54±2.99	2.580	0.077	1.63±2.72	2.606	0.075
	No	295	6.39±2.46			0.79±2.06		
	I'm not sure	19	7.05±2.48			1.15±1.89		
**The amount of smoking during the pandemic**	Increased	91	1.30±2.59	27.077	**<0.001**	13.00±10.68	4.529	**0.011**
	Decreased	35	1.46±3.12			20.88±13.27		
	Unchanged	223	0.64±1.67			17.64±10.64		
	Sidak *post hoc* test		**1–2 p<0.001**			**1–3 p=0.030**		
			**1–3 p=0.005**					
			**2–3 p<0.001**					
**When did you decide to quit smoking?**	Before the epidemic began	182	6.35±2.55	0.473	0.624	0.7±1.81	2.007	0.136
	When the epidemic began	23	5.87±3.09			1.52±2.89		
	After the epidemic	144	6.42±2.42			1.03±2.37		
**Do you think the COVID-19 pandemic has an impact on smoking cessation?**	Yes	131	6.28±2.85	-0.367	0.728	1.60±2.96	4.188	**<0.001**
	No	218	6.39±2.33			0.46±1.27		

t: independent samples test. F: one way ANOVA.

### Results of linear regression analysis between CAS total score and some variables

Table 3 shows the results of the multiple linear regression analysis performed to determine the variables predicting the total score of CAS. The results of the established model were statistically significant [F (5;343)=8.89, p<0.001]. The total score of CAS was 0.70 points higher in females than in males (β=0.70; 95% CI: 0.25–1.15, p=0.002). The total CAS score was 1.04 points higher among those who said that the COVID-19 pandemic had an impact on quitting smoking than among those who said it did not [β=1.04; 95% CI: 0.59–1.48, p<0.001]. The total score of CAS was 0.55 points higher among those who said the amount of smoking increased during the pandemic than among those who said it had decreased (β=0.55; 95% CI: 0.59–1.05, p=0.028). The variables ‘How did your smoking amount change during the pandemic?’ (β=0.71; p=0.054) and ‘Are there any COVID-19 positive people around the participants?’ (β=-0.21, p=0.236) had no significant effect on the total score of CAS. It was found that the 11% variance in CAS score could be explained by gender and the variables ‘Does the COVID-19 pandemic have an effect on quitting smoking?’ and ‘The amount of smoking in the epidemic’ (R^2^=0.11).

**Table 3:** Results of linear regression analysis between CAS total score and some variables (N=349)

	*Unstandardized β*	*p*	*95% CI*
Constant	0.37	0.270	-0.29–1.03
Gender (Ref: male)	0.70	**0.002**	0.25–1.15
Do you think the COVID-19 pandemic has an impact on smoking cessation? (Ref: no)	1.04	**<0.001**	0.59–1.48
The changes in the amount of smoking during the pandemic? (Ref: decreased)	0.55	**0.028**	0.59–1.05
The change in the amount of smoking during the pandemic? (Ref: increased)	0.71	0.054	-0.14–1.44
Are there any COVID-19 positive people around you? (Ref: no)	-0.21	0.236	-0.56–0.13

R=0.33; R^2^=0.11; F=8.89; p<0.001.

### Comparison of knowledge questions with different variables

As can be seen in Table 4, the impact of the COVID-19 pandemic on smoking cessation was statistically different between knowing that the rate of intensive care unit (ICU) admissions due to COVID-19 infection is higher in smokers, that smokers are heavily infected with COVID-19 and that the mortality rate due to COVID-19 infection is higher in smokers (p<0.001)

**Table 4:** Comparison of knowledge questions with different variables (N=349)

*Variables*	*Categories*	*Smokers are heavily infected with COVID-19*			*The rate of intensive care unit (ICU) admissions due to COVID-19 infection is higher in smokers*			*The mortality rate due to COVID-19 infection is higher among smokers*		
		*Wrong/no answer*	*Right*	*p*	*Wrong/no answer*	*Right*	*p*	*Wrong/no answer*	*Right*	*p*
**Age** (years)	≤20	4 (36.4)	7 (63.6)	0.909	3 (27.3)	8 (72.7)	0.795	3 (27.3)	8 (72.7)	0.205
	21–30	45 (42.5)	61 (57.5)		44 (41.5)	62 (58.5)		46 (43.4)	60 (56.6)	
	31–40	46 (42.6)	62 (57.4)		45 (41.7)	63 (58.3)		45 (41.7)	63 (58.3)	
	41–50	36 (39.1)	56 (60.9)		34 (37.0)	58 (63.0)		34 (37.0)	58 (63.0)	
	>50	11 (34.4)	21 (65.6)		11 (34.4)	21 (65.6)		7 (21.9)	25 (78.1)	
**Gender**	Male	52 (41.9)	72 (58.1)	0.725	52 (41.9)	72 (58.1)	0.446	56 (45.2)	68 (54.8)	0.065
	Female	90 (40.0)	135 (60.0)		85 (37.8)	140 (62.2)		79 (35.1)	146 (64.9)	
**Education level**	Primary school or literate	32 (36.4)	56 (63.6)	0.564	34 (38.6)	54 (61.4)	0.389	32 (36.4)	56 (63.6)	0.773
	Middle/high school	74 (44.6)	92 (55.4)		72 (43.4)	94 (56.6)		69 (41.6)	97 (58.4)	
	Undergraduate degree	32 (38.1)	52 (61.9)		27 (32.1)	57 (67.9)		30 (35.7)	54 (64.3)	
	Postgraduate degree	4 (36.4)	7 (63.6)		4 (36.4)	7 (63.6)		4 (36.4)	7 (63.6)	
**Marital status**	Married	95 (39.6)	145 (60.4)	0.756	90 (37.5)	150 (62.5)	0.393	89 (37.1)	151 (62.9)	0.661
	Single	38 (42.2)	52 (57.8)		37 (41.1)	53 (58.9)		38 (42.2)	52 (57.8)	
	Widowed/ divorced	9 (47.4)	10 (52.6)		10 (52.6)	9 (47.4)		8 (42.1)	11 (57.9)	
**Chronic disease**	Present	28 (33.7)	55 (66.3)	0.140	25 (30.1)	58 (69.9)	0.051	26 (31.3)	57 (68.7)	0.115
	Absent	114 (42.9)	152 (57.1)		112 (42.1)	154 (57.9)		109 (41)	157 (59)	
**Regular use of medication**	Present	37 (35.9)	66 (64.1)	0.241	35 (34)	68 (66)	0.192	35 (34.0)	68 (66.0)	0.243
	Absent	105 (42.7)	141 (57.3)		102 (41.5)	144 (58.5)		100 (40.7)	146 (59.3)	
**Are there any COVID-19 positive people around you?**	Yes	69 (42.9)	92 (57.1)	0.664	63 (39.1)	98 (60.9)	0.405	62 (38.5)	99 (61.5)	0.638
	No	63 (38.2)	102 (61.8)		62 (37.6)	103 (62.4)		62 (37.6)	103 (62.4)	
	I'm not sure	10 (43.5)	13 (56.5)		12 (52.2)	11 (47.8)		11 (47.8)	12 (52.2)	
**If there is a COVID-19 positive person around you, where?**	At home in my family	31 (39.7)	47 (60.3)	0.455	32 (41)	46 (59)	0.851	31 (39.7)	47 (60.3)	0.713
	At the workplace	17 (40.5)	25 (59.5)		15 (35.7)	27 (64.3)		14 (33.3)	28 (66.7)	
	Other	21 (51.2)	20 (48.8)		16 (39)	25 (61)		17 (41.5)	24 (58.5)	
**Have you had a COVID-19 infection?**	Yes	17 (48.6)	18 (51.4)	0.062	16 (45.7)	19 (54.3)	0.145	15 (42.9)	20 (57.1)	0.813
	No	113 (38.3)	182 (61.7)		110 (37.3)	185 (62.7)		112 (38)	183 (62)	
	I'm not sure	12 (63.2)	7 (36.8)		11 (57.9)	8 (42.1)		8 (42.1)	11 (57.9)	
**The amount of smoking during the pandemic**	Increased	40 (44)	51 (56)	0.031	38 (41.8)	53 (58.2)	0.110	41 (45.1)	50 (54.9)	0.072
	Decreased	7 (20)	28 (80)		8 (22.9)	27 (77.1)		8 (22.9)	27 (77.1)	
	Unchanged	95 (42.6)	128 (57.4)		91 (40.8)	132 (59.2)		86 (38.6)	137 (61.4)	
**When did you decide to quit smoking?**	Before the epidemic began	80 (44)	102 (56)	0.336	75 (41.2)	107 (58.8)	0.715	81 (44.5)	101 (55.5)	0.064
	When the epidemic began	7 (30.4)	16 (69.6)		8 (34.8)	15 (65.2)		8 (34.8)	15 (65.2)	
	After the epidemic	55 (38.2)	89 (61.8)		54 (37.5)	90 (62.5)		46 (31.9)	98 (68.1)	
**Do you think the COVID-19 pandemic has an impact on smoking cessation?**	Yes	28 (21.4)	103 (78.6)	<0.001	28 (21.4)	103 (78.6)	**<0.001**	27 (20.6)	104 (79.4)	**<0.001**
	No	114 (52.3)	104 (47.7)		109 (50)	109 (50)		108 (49.5)	110 (50.5)	
**Nicotine dependence according to FTND**	Very mild	12 (37.5)	20 (62.5)	0.756	12 (37.5)	20 (62.5)	0.382	12 (37.5)	20 (62.5)	0.422
	Mild	21 (43.8)	27 (56.3)		19 (39.6)	29 (60.4)		15 (31.3)	33 (68.8)	
	Moderate	20 (48.8)	21 (51.2)		22 (53.7)	19 (46.3)		21 (51.2)	20 (48.8)	
	Severe	37 (37.4)	62 (62.6)		36 (36.4)	63 (63.6)		38 (38.4)	61 (61.6)	
	Extremely severe	52 (40.3)	77 (59.7)		48 (37.2)	81 (62.8)		49 (38)	80 (62)	

Data are given as n (%), where percentages are for right or wrong/no answer. Pearson chi-squared test. FTND: Fagerström test for nicotine dependence.

## DISCUSSION

During epidemics, the number of people whose mental health is affected tends to be higher than the number of people affected by the infection^21^, with fear and stress as widespread strong emotions^22^. During a pandemic, fear increases anxiety levels in healthy people and exacerbates symptoms of pre-existing psychiatric disorders^23^. Several studies have found that anxiety levels in the general population have been high since the beginning of the COVID-19 era^24^. The study of more than 1200 people in China showed that 29% of participants suffered from moderate to severe anxiety during the pandemic^25^.

Healthcare workers, in particular, struggle with psychological problems such as predominantly fear and anxiety during a pandemic. In the study of Lu et al.^26^, employees of departments that have close contact with patients and are exposed to high levels of aerosols have a 2-fold higher risk of suffering from fear, anxiety, and depression than employees of departments that have relatively little contact and exposure to aerosols.

A study on stress perception of Italian athletes revealed that male athletes had lower stress perception than female athletes during the COVID-19 quarantine^27^. Our study also revealed that fear of COVID-19 was higher in female participants. There are many studies showing that the increased stress of a disease with high morbidity and mortality, such as COVID-19, causes an increase in negative emotions such as hopelessness, anxiety, fear of infection due to isolation and curfews, which are among the measures taken, and changes in smoking behavior^28-30^. It was observed that the pandemic caused some smokers to increase smoking while other smokers reduced smoking^31-35^.

The association between the high mortality and morbidity rates in people infected with COVID-19 and smoking can be seen as a motivating factor for people to quit smoking. Our study found that about a quarter of the participants increased their smoking amount during the pandemic. In a study conducted with 1359 smokers in the US, about half of the smokers reported that their smoking amount had not changed during the pandemic, whereas 22% reported smoking more^36^. However, a study from the United Kingdom showed that a quarter of smokers smoked more during the pandemic and that mental health and psychosocial well-being were strongly related to tobacco use^37^.

The increase in smoking amount during the pandemic among those with higher COVID-19 anxiety levels in our study supports the finding of the UK study. The literature suggests that some nicotine withdrawal symptoms may resemble the physiological stress response and contribute to an increase in stress-related smoking cravings^38^. It is also suggested that nicotine dependence is the initial reason for the continuation of smoking behavior and overall failure of treatment attempts^39^. In a study conducted by Örsel et al.^40^, 33% of patients presenting to the smoking cessation polyclinic were highly dependent on nicotine. More than two-thirds of the participants were considered extremely nicotine dependent in our study, which could explain the increase in smoking during the pandemic and only half of the decrease in smoking cessation and smoking amount among those who were examined.

We observed that those who had increased smoking during the pandemic were more stressed and more dependent. We can explain this result by the fact that people, especially those with high addiction potential, turn to addictive substances to relieve their negative feelings, as found in one study^41^. In our study, we determined that those who thought that the COVID-19 pandemic had an impact on smoking cessation and that they knew that the rate of ICU hospitalization and death was higher among smokers infected with COVID-19 had decreased the amount of cigarette or hookah smoking.

### Limitations

The limitations of the study are that it only measures the level of anxiety in smokers who visited our smoking cessation outpatient clinic during the pandemic, that it measures the effect of COVID-19 on smoking behavior only with the level of anxiety, and that it does not address the frequency of smoking and anxiety level in the community during the pandemic. In addition, the small number of participants and the fact that it was conducted in a single center are other limitations of the study. On the contrary, the limited number of studies on the association between the level of anxiety due to COVID-19 and smoking can be considered as a strength of our study. Future studies will better illuminate the long-term effects of this pandemic on smoking behavior by examining the smoking status of those who quit smoking or those who never smoked during the pandemic.

## CONCLUSIONS

Pandemic-related anxiety is a factor in the decision to quit smoking. It has been observed that individuals who have high levels of pandemic anxiety due to COVID-19 quit smoking and reduced the number of cigarettes they smoked at the onset of the pandemic. The level of anxiety related to COVID-19 is higher among females and individuals who smoke during the pandemic. The knowledge that COVID-19 infection is severe in smokers is one of the effective factors in the decision to quit smoking during the pandemic. We will not be able to thoroughly examine the factors that influence pandemic-related anxiety until the pandemic is over. What we need to do in the current pandemic is to intervene early in patients who develop anxiety symptoms and turn this situation into an opportunity for healthy behaviors such as eliminating harmful habits like tobacco addiction. In the time of the pandemic, we need more than ever training for health professionals, both for the fight against the pandemic and for preventive and curative healthcare.

## Data Availability

The data supporting this research are available from the authors on reasonable request.
